# Abstracta

**Published:** 1885-07

**Authors:** 


### Abstracta.

"A girl has been arrested while disguised as an old woman. The old woman disguised as a girl is still at large."

"Colored Woman—"Boss, kin I get de job ob cleanin' out dis heah bank?" President—"No! You are too late. The cashier has already attended to that."—*Texas Siftings*.

WELL BEGUN IS HALF DONE.—A young pupil who was obliged to give an illustration to his teacher of the above motto seemed puzzled when his effort. "He that is well lathered is half shaved"-produced a series of audible smiles.

"When we are in the company of sensible men we ought to be doubly cautious of talking too much, lest we lose two good things—their good opinion and our own improvement; for what we have to say we know. but what they have to say we know not."

THE DIFFERENCE.—Schoolboy, Decatur, Ga.: Is there any difference between a journalist and an editor? Yes. The journalist is a man who writes things for newspapers. The editor is the man who leaves out what the journalist writes. —*Atlanta Constitution*.

AN INTERESTING CASE.—"Three medical celebrities meet together to consult at the sick-bed of General X. After they go, the General rings for his man servant." "Well, John, you showed those gentlemen out; what did they say?" "Ah, General they seem to differ with each other. The big, fat one said that they must have a little patience, and at the autopsy—whatever that may be-they would find out what the matter was."

—The wife of an optical instrument maker on landing at New York after a European tour, attempted to smuggle under her dress a quantity of artificial eyes. In reply to the usual question whether she had anything to declare, she said "No." most postively; but on the officer shaking her dress, the deception was exposed, and, in spite of her "Nos," the eyes had it. But how absurd of the fair smuggler to hope to escape detection when every eye was upon her! —*Exchange*.

Who loves his work and knows how to spare, may live and flourish anywhere.

"Which is the most intelligent, the man who knows most, or the one who has most nose?"

"A lady of a certain age says that the reason an old maid is generally so devoted to her cat is that, not having any husband she naturally takes the next most treacherous animal."

SIZE OF THE EAR AS AN INDICATION OF GOOD CHARACTER.—A physician in Detroit says that a small ear is an indication of bad character and that criminals have the lobule of the ear imperfectly developed. What a powerful good fellow the jackass must be.

(1) "An humble boy, with shining pail, went gladly singing adown the dale, to where the cow with the brindle tail on clover her palate did regale. An humble bee did gayly sail far over the soft and shadowy vale, to where the boy with the shining pail was milking the cow with the brindle tail.

(2) The bee sat down on the cow's left ear; her heels flew up through the atmosphere—and through the leaves of the chestnut bough the boy is cheerily soaring now."

ABSURDITIES OF HUMAN LIFE.—Not to go to bed when you are sleepy because it is not a certain hour.

To stand in water up to your knees fishing for trout, when you can buy them in a clean, dry market.

People of exquisite sensibility, who cannot bear to see an animal put to death, showing the utmost attention to the variety and abundance of their tables.

The heir of an avaricious uncle paying him the compliment of the deepest mourning.

The lovely widow of a cross old man wearing weeds; and the survivor of a rich old shrew being particular in the choice and display of his weepers.

To buy a horse from a near relation, and believe every word he says in praise of the animal he is desirous to dispose of.

To call a man hospitable who indulges his vanity by displaying his service of plate to his rich neighbors frequently, but was never known to give a dinner to any one really in want of it. —*Ex.*

